# Gender Differences in Concerns About Participating in Cancer Research During the COVID-19 Pandemic

**DOI:** 10.1177/1073274821989315

**Published:** 2021-01-25

**Authors:** Louis Fox, Harriet Wylie, Fee Cahill, Anna Haire, Saran Green, Joyce Kibaru, Catherine Hartley, Richard Sullivan, Mieke Van Hemelrijck

**Affiliations:** 1 Translational Oncology and Urology Research (TOUR), School of Cancer and Pharmaceutical Sciences, King’s College London, United Kingdom; 2 Ecancer, Bristol, United Kingdom; 3 Institute of Cancer Policy, King's College London, United Kingdom

**Keywords:** SARS-CoV-2, coronavirus, gender, oncology, engagement, anxiety

## Abstract

**Introduction:**

The ongoing SARS-CoV-2 pandemic is having major effects on cancer research, including major reductions in participant accrual to cancer clinical trials. Existing research has indicated that these steep drops in accrual rates to cancer clinical trials may be disproportionately affecting women. We sought to determine if there were gender differences in a dataset collected to examine participants’ concerns about taking part in cancer research during the pandemic.

**Methods:**

Between 5-19 June 2020, we distributed a fully anonymized survey via social media. We contacted 85 UK cancer patient organizations/charities and asked them to share our questionnaire on their platforms, of which 26 obliged. Patients aged 18 with a cancer diagnosis were eligible to participate and asked about their clinical and demographic characteristics, concerns about research participation given the COVID-19 pandemic, anxiety levels measured using the Generalized Anxiety Disorder-7 (GAD-7) scale, amongst other questions. Anxiety levels and concerns about participating were compared between men and women using univariate and multivariate analyses.

**Results:**

93 individuals, comprising n = 37 women and n = 56 men of various cancer types, provided survey responses. Independent t-tests showed that women reported higher anxiety scores, and concerns about participating in cancer research during COVID-19, than men. Linear regression analyses showed that anxiety scores predicted concerns about research participation in women but not men (p_interaction_ = 0.002).

**Conclusions:**

Cancer patients have concerns about participating in research during the COVID-19 pandemic that range from mild to serious. Furthermore, the relationship between general anxiety and concerns about research participation may be both more relevant and more pronounced in women than in men. Future work should examine the reasons why women are less likely to enrol in cancer trials during the COVID-19 pandemic.

## Introduction

The ongoing SARS-CoV-2 pandemic is continuing to inflict a severe public health challenge throughout the world.^1^ As of October 2020, there were more than 42 million confirmed cases of infection with SARS-CoV-2 globally, and upward of 1.1 million fatalities from the resulting disease, COVID-19.^2^ The ease via which the SARS-CoV-2 virus is transmitted, combined with the rates of hospitalization for vulnerable populations (elderly and comorbid), has meant that healthcare systems have been significantly disrupted by the outbreak.^3^

It is evident that the provision of cancer services has been strongly impacted by the outbreak of SARS-CoV-2.^4^ Suspected cancer referrals have declined sharply,^5^ creating a concern that many individuals could be missing an opportunity for curative treatment.^4^ Furthermore, clinical guidelines are yet to be uniformly established for the management of cancer patients in a SARS-CoV-2 epidemic.^6^

Whilst work to address issues related to the immediate provision of cancer care is extremely urgent, it is also important to examine the impact that the pandemic may be having on cancer research, particularly that involving patients.^7,8^ Governmental agencies and other research funders such as charities have been issuing guidance regarding clinical trials during the pandemic,^9-11^ and research teams have been adapting, with some reporting successful clinical trials continuity.^12-15^ However, there remains an important question with regard to the impact that the COVID-19 pandemic may be having on participation in cancer research by cancer patients and survivors.

A cohort study conducted by Unger et al has shown that participation in cancer clinical trials in the Southwest Oncology Group (SWOG) Cancer Research Network in the United States dropped by approximately 50% following the outbreak of SARS-CoV-2 in the USA in March 2020.^16^ It should be noted that accrual rates to such studies were almost certainly affected by mitigation measures enacted by participating healthcare centers to respond to a local SARS-CoV-2 outbreak. However, it can also be hypothesized that anxieties surrounding the outbreak of SARS-CoV-2 may have resulted in increased hesitancy on the part of cancer patients and survivors to participate in research studies, or increased hesitancy on the part of clinicians to enrol patients in trials. Analysis of this cohort by Unger and colleagues showed that decreases in trial accrual rates coinciding with the outbreak of SARS-CoV-2 were significantly more pronounced in women than in men (despite seeing no such differences across age groups or ethnicities).^16^

We recently conducted a small preliminary survey of cancer patients, that was designed to provide some initial insights into the potential concerns of people with cancer about participating in research during the SARS-CoV-2 pandemic. We set out to rapidly collect data from a broad sample of cancer types, that could provide some preliminary indications of areas that should be subjected to further scientific scrutiny in future, more focused studies. For our part, the data will inform a qualitative study examining cancer patients’ concerns about participating in cancer research studies during the SARS-CoV-2 pandemic.^17^ The preliminary survey collected information on cancer patients’ concerns about participating in cancer research during the pandemic, as well as information on clinical and demographic characteristics and current anxiety levels. Acknowledging the gender disparities in cancer study accrual observed by Unger et al, we examined this data to discern if there were gender differences in our sample with regard to concerns about participating in cancer research during the pandemic.

## Materials and Methods

Anyone aged 18 and above with a cancer diagnosis of any type was eligible to participate in this cross-sectional survey. Participants were recruited via social media (Twitter and Facebook). Relevant charities, cancer-specific patient support groups, cancer research groups and patient research reference groups were contacted and asked if they would agree to distribute a questionnaire link on either their website, Twitter, or Facebook. We contacted 85 organizations, of which 26 agreed to distribute the survey. One hundred and twenty-six individuals consented to participate in the study. One hundred and one of these individuals provided complete survey responses (80%). It was apparent that 93% of respondents (n = 93) were from the UK; hence 8 non-UK respondents were omitted from the analysis for the purposes of homogeneity, creating a final sample of 93 UK based participants.

Individuals who followed the URL to the survey were presented with a participant information page, which provided potential participants with details of the nature of the questionnaire and all other information necessary for informed consent, i.e. regarding ethics approval, study anonymity, and right to withdraw. The online questionnaire, which was compiled ad hoc by our group (apart from the anxiety questions), queried: 1) clinical and demographic characteristics; 2) individual circumstances (e.g. whether the respondent lives alone, how they travel to their appointments, whether they have care responsibilities, etc.); 3) self-reported likelihood of not participating in cancer research due to the pandemic; 4) concerns about participating in cancer research, relating to COVID-19; and 5) current anxiety levels. A copy of the questionnaire is available in Appendix A.

The questionnaire asked participants how comfortable they would feel about participating in a cancer research study during the COVID-19 pandemic, with multiple choice responses clearly implicating COVID-19 as the reason for potentially not participating (for example, *“I would have concerns due to Covid-19 that I would need addressing/resolving by study researchers, before I could participate”*; or *“I would have concerns due to Covid-19, and would not participate for that reason”*).

The questionnaire presented participants with a response matrix asking them about specific potential concerns relating to COVID-19, that might affect their likelihood of participating in cancer research. This question was phrased *“Please indicate how concerning each of the following things are to you, with regard to taking part in cancer research during the current Covid-19 pandemic.”* Participants indicated their level of concern about each specific category using a 4-point Likert scale, consisting of “Not at all concerned,” “Mildly concerned,” “Moderately concerned,” and “Seriously concerned.” The concerns listed were: *“My age”*; *“My (non-cancer) medical conditions”*; *“My occupation/job”*; *“My ethnicity/race”*; *“Financial or health insurance concerns”*; *“The type of research study that I am taking part in”; “Having to travel somewhere to take part in research”; “The type of travel that I have to use to take part (e.g. bus, car, train)”*; *“Those that I live with”*; *“Those that I have caring responsibilities toward (not including childcare)”*; *“Cancer treatment that I am currently undergoing”*; *“Cancer treatment that I have previously undergone”*; *“The type of cancer that I have been diagnosed with”*; and *“Other concerns.”* Responses to these questions were quantified (0-3 for each concern) and summed to create a “Total concerns” score between 0 to 39, in which a higher score indicated more concerns about research participation overall.

In addition to the above questions, general state of anxiety was measured using the Generalized Anxiety Disorder-7 (GAD-7).^18^ The GAD-7 asks respondents 7 questions relating to how they have felt in the past 2 weeks, resulting in an anxiety score ranging from 0 to 21 (0-9 = none to mild; 10-14 = moderate; 15-21 = severe).

An analysis was conducted to examine:

1. associations between concerns about research participation, and self-reported likelihood of not participating in cancer research due to COVID-19
2. whether there were gender-related differences in self-reported likelihood of not participating in cancer research due to COVID-19
3. whether there were gender-related differences in overall concerns about participating, related to COVID-19 (i.e. “Total concerns” score)
4. whether there were gender-related differences in current anxiety levels
5. whether anxiety levels were associated with concerns about participating, both in the sample as a whole and within each gender category

We examined these research questions using Kruskal-Wallis tests of association between specific concerns about participating during the pandemic, and self-reported likelihood of nonparticipation due to COVID-19. For the gender-based research questions, we used Chi-square tests to compare gender distributions for the categorical variables (*Self-reported likelihood of nonparticipation due to COVID-19*); independent t-tests to compare genders for the continuous variables (*Total concerns* and *Current anxiety*); and linear regression models to examine the relationships between gender, *Current anxiety*, and *Total concerns*. We also examined whether there were gender differences in treatment types received, to account for the fact that individuals receiving immunocompromising treatments may report more anxieties and concerns about participating.

The study was approved by the Research Ethics Office at King’s College London (Ethical Clearance Reference Number: MRA-19/20-19372).

## Results

Responses from 93 cancer patients were included in the analysis (Table 1). Thirty-seven (39.8%) of respondents were female and fifty-six (60.2%) respondents were male. Most of the sample were of White ethnicity (93.5%). The median age category was 60-69 years. Prostate cancer patients comprised 40% of the sample, followed by 23% colorectal, 12% breast, 8% bladder, 8% kidney cancer, and 11% other cancers. Most respondents reported their most recently treatment as either chemotherapy (21.5%), surgery (19.4%), or hormone therapy (25.8%). Interestingly, 57% of participants had never previously participated in research.

**Table 1.** Description of Sample.

	Female		Male		Total	
	n	(%)	n	(%)	n	(%)
	37	(39.8)	56	(60.2)	93	-
**Ethnicity**						
White	35	(97.2)	52	(92.9)	87	(93.5)
Black (Caribbean descent)	0	(0)	2	(3.6)	2	(2.2)
South Asian	0	(0)	1	(1.8)	1	(1.1)
Mixed ethnicity	1	(2.8)	1	(1.8)	2	(2.2)
**Age**						
30-39	6	(16.2)	2	(3.6)	8	(8.6)
40-49	5	(13.5)	4	(7.1)	9	(9.7)
50-59	11	(29.7)	8	(14.3)	19	(20.4)
60-69	11	(29.7)	23	(41.1)	34	(36.6)
70-79	4	(10.8)	18	(32.1)	22	(23.7)
80-89	0	(0)	1	(1.8)	1	(1.1)
**Cancer type**						
Bladder	2	(5.4)	5	(8.9)	7	(7.5)
Blood	1	(2.7)	1	(1.8)	2	(2.2)
Breast	11	(29.7)	0	(0)	11	(11.8)
Colorectal	14	(37.8)	7	(12.5)	21	(22.6)
Gynaecological	2	(5.4)	0	(0)	2	(2.2)
Head & neck	0	(0)	2	(3.6)	2	(2.2)
Kidney	4	(10.8)	3	(5.4)	7	(7.5)
Liver	0	(0)	1	(1.8)	1	(1.1)
Lung	1	(2.7)	0	(0)	1	(1.1)
Lymphoid	0	(0)	1	(1.8)	1	(1.1)
Prostate	0	(0)	37	(64.3)	37	(39.8)
Skin (melanoma)	1	(2.7)	0	(0)	1	(1.1)
**Most recent treatment**						
Chemotherapy	13	(35.1)	7	(12.5)	20	(21.5)
Chemoradiotherapy	2	(5.4)	2	(3.6)	4	(4.3)
Surgery	7	(18.9)	11	(19.6)	18	(19.4)
Radiotherapy/brachytherapy/HIFU	1	(2.7)	5	(8.9)	6	(6.5)
Hormone therapy	5	(13.5)	19	(33.9)	24	(25.8)
Immunotherapy	4	(10.8)	4	(7.1)	8	(8.6)
Active surveillance	3	(8.1)	3	(5.4)	6	(6.5)
Other	2	(5.4)	5	(8.9)	7	(7.5)
**Currently undergoing cancer treatment?**						
No	13	(35.1)	23	(41.1)	36	(38.7)
Yes	24	(64.9)	33	(58.9)	57	(61.3)
**Participation in cancer research**						
Never have	24	(64.9)	29	(51.8)	53	(57)
Participated previously	11	(29.7)	15	(26.8)	26	(28)
Participated previously, withdrew due to COVID-19	0	(0)	1	(1.8)	1	(1)
Currently participating	2	(5.4)	11	(19.6)	13	(14)

Participants who reported higher concerns about their age, having to travel to participate, previous cancer treatment they have received, and the type of cancer they had been diagnosed with, were more likely to report that they would not participate—or would need concerns addressing by researchers before participating—due to COVID-19 (see Table 2).

**Table 2.** Frequency Distributions (With Percentages) of Levels of Specific Concerns About Taking Part in Cancer Research During the SARS-CoV-2 Outbreak.

	Not at all concerned	Mildly concerned	Moderately concerned	Seriously concerned	Association with self-reported likelihood of nonparticipation due to COVID-19
	n (%)				p
Age	53 (57.0)	21 (22.6)	13 (14.0)	6 (6.5)	0.016
Non-cancer medical conditions	58 (62.4)	13 (14.0)	18 (19.4)	4 (4.3)	0.942
Work	83 (89.2)	4 (4.3)	4 (4.3)	2 (2.2)	0.928
Ethnicity	92 (98.9)	1 (1.1)	0	0	0.624
Ethnicity (PoC only)	4 (80)	1 (20)	0	0	0.607
Financial/insurance	79 (84.9)	9 (9.7)	3 (3.2)	2 (2.2)	0.360
Type of research study	64 (68.8)	17 (18.3)	10 (10.8)	2 (2.2)	0.708
Having to travel	27 (29)	27 (29)	23 (24.7)	16 (17.2)	0.002
Mode of travel needed	28 (30.1)	23 (24.7)	22 (23.7)	20 (21.5)	0.327
Those that the participant lives with	58 (62.4)	19 (20.4)	11 (11.8)	5 (5.4)	0.933
Caring responsibilities (not childcare)	75 (80.6)	10 (10.8)	6 (6.5)	2 (2.2)	0.838
Ongoing cancer treatment	58 (62.4)	17 (18.3)	9 (9.7)	9 (9.7)	0.125
Previous cancer treatment	67 (72)	15 (16.1)	7 (7.5)	4 (4.3)	0.035
Type of cancer diagnosed	53 (57)	14 (15.1)	19 (20.4)	7 (7.5)	0.003
Other concerns	83 (89.2)	5 (5.4)	3 (3.2)	2 (2.2)	0.094

The table also presents p-values from kruskal-wallis tests examining associations between levels of concern on each issues, and self-reported likelihood of nonparticipation in cancer research due to COVID-19. PoC, people of color.

Females were more likely to report having received a potentially immunocompromising therapy as their most recent treatment (defined as chemotherapy, chemoradiotherapy, or immunotherapy) (p = 0.005). Following this observation, we examined whether individuals reporting receipt of immunocompromising treatment showed any differences in *Current anxiety*, *Total concerns*, or *Self-reported likelihood of non-participation due to COVID-19*, compared with those reporting other treatments. There were no significant differences between these 2 groups on any of these variables (*Current anxiety*: p = 0.47; *Total concerns*: p = 0.51; *Self-reported likelihood of non-participation due to COVID-19*: p = 0.32).

Although more females than males reported that they would be less likely to participate, or would not participate, in research due to COVID-19 (females: 27% vs. males: 14.3%), this was not statistically significant (p = 0.13).

In addition, it was found that females had significantly higher scores on *Total concerns* than males (females: M = 10.0, SD = 5.6 vs. males: M = 6.5, SD = 5.6, p = 0.004); a similar gender difference was seen for *Anxiety levels* as scored by the GAD-7 (females: M = 7.2, SD = 7.2 vs. males: M = 2.4, SD = 2.9, p < 0.001). In females, *Anxiety levels* were relatively high: 21.6% of females reported anxiety scores that the GAD-7 scoring system interprets as “severe” anxiety.

**Figure 1.**
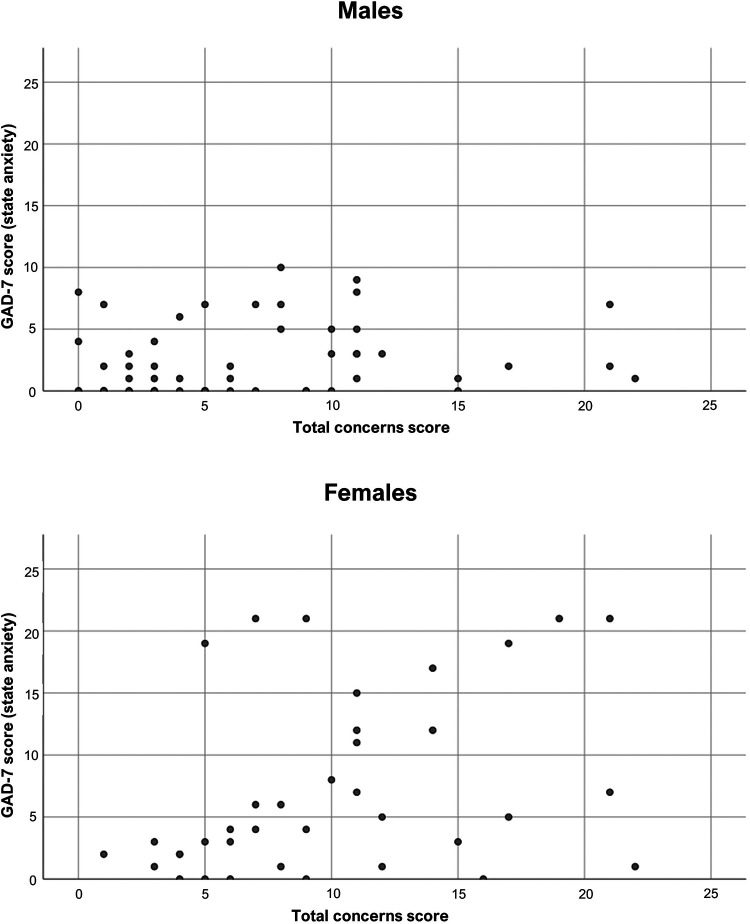
Scatterplots showing “Current anxiety” scores plotted against “Total concerns” about cancer research participation during the SARS-CoV-2 pandemic, for men and women respectively. GAD-7, Generalized Anxiety Disorder-7.

We then set out to conduct a test for interaction for the association between *Anxiety levels* and *Total concerns* as stratified analyses had shown that there was a statistically significant association for females but not for males (females: p = 0.027 vs. males: p = 0.137) (p_interaction_ = 0.002) (see Figure 1). These findings remained upon adjustment for immunocompromising treatment.

## Discussion and Conclusions

The data analyzed here provides a preliminary indication that individuals with cancer in the United Kingdom have concerns about participating in cancer research during the SARS-CoV-2 pandemic, that range from “mild” to “serious.” Concerns reported in this sample that were associated with self-reported higher likelihood of nonparticipation due to COVID-19 were centered around the individual’s age, the need to travel to participate, previous cancer treatments received, or concerns related to the specific type of cancer they have been diagnosed with. Anxiety levels were associated with concerns about participating in general. However, the nature of the relationship between state anxiety levels, and concerns about participating in cancer research during the pandemic, was significantly modified by gender.

To our knowledge, this is the first study to present data on attitudes toward participating in cancer research during the SARS-CoV-2 pandemic. Understanding these issues is likely to help cancer research teams to mitigate the impact that the pandemic is having on participant accrual. Although preliminary, these data highlight key areas that investigators may wish to examine further, perhaps qualitatively. It is particularly noteworthy that there appears to be gender disparities in anxiety levels during the pandemic that may be associated with hesitancy on the part of women to participate in cancer research studies. If this is the case, then without intervention the pandemic may exacerbate existing systemic gender biases in health research,^19^ which will serve to disadvantage women in the long term.

Our findings were suggestive that individual perceptions of risk (e.g. associated with age or having cancer) could have implications for cancer trial recruitment. Psychological research has shown that that risk perception is often subjective and cognitively biased.^20^ In terms of SARS-CoV-2, our findings are consistent with a survey that has been conducted on 1,300 people in Germany (preprint), which found no significant differences between older and younger people in fear of being infected with SARS-CoV-2,^21^ despite elderly people being at much higher risk of fatality following infection.^22^ It is possible that hesitancy to participate in cancer research during the SARS-CoV-2 epidemic could be driven by erroneous perceptions of risk, possibly linked with the perception that the authorities are not adequately protecting the public.^23^ Encouragingly, the same survey cited the most common coping strategy as “listening to experts and following their advice,” and 76.2% of respondents trusted clinical personnel to protect them.^21^ Supportive communication addressing individuals’ overestimation of the risk of taking part in cancer research could facilitate study recruitment.

A few studies have reported higher rates of anxiety among women than men during the SARS-CoV-2 pandemic.^24-27^ A shared caveat of these studies is that they all examine anxiety prevalence during the pandemic, but do not report comparative data against which it can be inferred that the pandemic is the driver of the anxiety prevalence reported. However, many studies in developmental psychology have shown that the nature in which women tend to psychologically respond to external threats differs to that of men,^28^ and so gender differences in this regard may perhaps be expected. A review of gender differences in fear and anxiety has documented how gender socialization processes during development, driven by sociocultural norms, may promote coping styles in women that are less effective at reducing anxieties provoked by perceived external threats.^28^ There is also some evidence that women are more likely to overestimate the probability of danger, and to expect harm, than men are.^29,30^

It is also important to acknowledge that more immediate social factors may be influencing anxieties about SARS-CoV-2. It has been reported that more than two-thirds of caregivers are daughters and wives.^31^ Given that it is well-established (and frequently communicated to the public) that the mortality of COVID-19 is dramatically higher in elderly people as compared with relatively younger adults,^32^ it could be the case that during the pandemic, women have generally been more likely to be in regular close contact with an elderly or unwell person for whom they provide care, and therefore they have concerns about the risks posed to others around them by SARS-CoV-2 transmission. Our questionnaire did ask participants if they have any care responsibilities, but we did not receive enough affirmative responses to this question to be able to investigate this hypothesis further.

Another key question would be to examine the relative degrees to which the gendered impact of the pandemic on research participant accrual is being driven by women not wishing to participate; or by clinicians/clinical researchers being less likely to invite women to participate. Unger et al have recently identified this as a key distinction to acknowledge in determining demographic disparities in trial accrual rates.^33^ Although our data showed that almost twice as many women than men reported that they would not necessarily participate in a research study due to COVID-19, this observation had limited statistical power and was not significant.

Initiatives already appear to be underway which may help to address some cancer patient concerns about participating in research, such as traveling to hospital. The impact that SARS-CoV-2 pandemic has inflicted on clinical trials has already resulted in efforts to minimize the amount of hospital visits required from participants at many centers,^14,15^ and some trialists have offered advice on how to achieve this safely.^34^ Some are now arguing that oncology research has historically required travel requirements from participants that are not necessary scientifically mandated, and that a more permanent shift toward alleviation of unnecessary burden on participants is now vital.^14,34,35^ The analysis we have reported here further supports these arguments. The increased adoption of telemedicine in oncology as a result of the pandemic^36^ may have a significant role to play in this regard, providing dual advantages of both safety and reassurance for participants, and the elimination of distance as a prohibiting factor to study recruitment (for some studies). Indeed, the qualitative study that we are conducting based on this analysis is making full use of remote meetings to interview participants.

Some limitations to this study should be considered. The sample lacked ethnic diversity—it is possible that Black and Asian cancer patients may have particular anxieties and concerns about the pandemic, given that data has indicated that these subpopulations may be more vulnerable to mortality from COVID-19.^37^ The sample also potentially lacked sensitivity to the issues concerning patients with specific cancer types, due to the broad spread of cancer types in the sample and the relatively small sample size.

As the logistic and economic impacts of SARS-CoV-2 on cancer research continue to unfold, it is important to ensure that the pandemic does not compound these issues by also affecting cancer research participation, particularly among women. Understanding the factors behind hesitancy to participate, including gender disparities in this regard, may help to mitigate this. As outlined above, future work in this area may wish to examine the relative extents to which the decline in cancer research participation among women during the pandemic are due to: i) individual psychological processes; ii) social/environmental factors; or iii) access to research studies.

## Supplemental Material

Supplemental Material, sj-pdf-1-ccx-10.1177_1073274821989315 - Gender Differences in Concerns About Participating in Cancer Research During the COVID-19 Pandemic Click here for additional data file. Supplemental Material, sj-pdf-1-ccx-10.1177_1073274821989315 for Gender Differences in Concerns About Participating in Cancer Research During the COVID-19 Pandemic by Louis Fox, Harriet Wylie, Fee Cahill, Anna Haire, Saran Green, Joyce Kibaru, Catherine Hartley, Richard Sullivan and Mieke Van Hemelrijck in Cancer Control

## References

[1] Sohrabi C Alsafi Z O’Neill N, et al. World Health Organization declares global emergency: a review of the 2019 novel coronavirus (COVID-19). Int J Surg. 2020;76:71–76. 32112977 10.1016/j.ijsu.2020.02.034 PMC7105032

[2] Worldometer. Coronavirus update (Live). Published 2020. Updated June 16, 2020. Accessed June 16, 2020. https://www.worldometers.info/coronavirus/

[3] Verelst F Kuylen E Beutels P. Indications for healthcare surge capacity in European countries facing an exponential increase in coronavirus disease (COVID-19) cases, March 2020. Eurosurveillance. 2020;25(13):2000323. 10.2807/1560-7917.ES.2020.25.13.2000323 PMC7140594 32265003

[4] The Lancet Oncology. Safeguarding cancer care in a post-COVID-19 world. Lancet Oncol. 2020;21(5):603. 32359483 10.1016/S1470-2045(20)30243-6 PMC7252123

[5] Lai AG Pasea L Banerjee A, et al. Estimating excess mortality in people with cancer and multimorbidity in the COVID-19 emergency. medRxiv. 2020:2020.05.27.20083287.

[6] Burki TK. Cancer guidelines during the COVID-19 pandemic. Lancet Oncol. 2020;21(5):629–630. 32247319 10.1016/S1470-2045(20)30217-5 PMC7270910

[7] Kutikov A Weinberg DS Edelman MJ Horwitz EM Uzzo RG Fisher RI. A war on two fronts: cancer care in the time of COVID-19. Ann Int Med. 2020;172(11):756–758. 32219410 10.7326/M20-1133 PMC7133056

[8] Hanna TP Evans GA Booth CM. Cancer, COVID-19 and the precautionary principle: prioritizing treatment during a global pandemic. Nat Rev Clin Oncol. 2020;17(5):268–270. 32242095 10.1038/s41571-020-0362-6 PMC7117554

[9] UK Medicines and Healthcare Regulatory Agency (MHRA). Managing clinical trials during coronavirus (COVID-19). Published 2020. Updated May 21, 2020. Accessed June 17, 2020. https://www.gov.uk/guidance/managing-clinical-trials-during-coronavirus-covid-19

[10] US Food and Drug Administration (FDA). FDA Guidance on Conduct of Clinical Trials of Medical Products During COVID-19 Public Health Emergency: Guidance for Industry, Investigators, and Institutional Review Boards. Published 2020. Updated December 4, 2020. Accessed June 19, 2020. https://www.fda.gov/media/136238/download

[11] European Medicines Agency (EMA). Guidance to sponsors on how to manage clinical trials during the COVID-19 pandemic. Published 2020. Updated March 20, 2020. Accessed June 19, 2020. https://www.ema.europa.eu/en/documents/press-release/guidance-sponsors-how-manage-clinical-trials-during-covid-19-pandemic_en.pdf

[12] Dipasquale A Persico P Lorenzi E Bertossi M Santoro A Simonelli M. Conducting phase I trials during the SARS-Coronavirus-2 outbreak: about science and care. Front Oncol. 2020;10:926. 32574280 10.3389/fonc.2020.00926 PMC7256652

[13] Moujaess E Kourie HR Ghosn M. Cancer patients and research during COVID-19 pandemic: a systematic review of current evidence. Crit Rev Oncol/Hematol. 2020;150:102972. 10.1016/j.critrevonc.2020.102972 PMC7174983 32344317

[14] Fontana E Arkenau H-T. Oncology clinical trials during the COVID-19 outbreak: lessons learnt during the crisis and future opportunities. Cancer Treat Rev 2020;88:102047. 32544543 10.1016/j.ctrv.2020.102047 PMC7286270

[15] Cancer Research UK. COVID-19: keeping patients on cancer clinical trials. Published 2020. Updated June 15, 2020. Accessed June 22, 2020. https://scienceblog.cancerresearchuk.org/2020/06/15/covid-19-keeping-patients-on-cancer-clinical-trials/

[16] Unger JM Blanke CD LeBlanc M Hershman DL. Association of the coronavirus disease 2019 (COVID-19) outbreak with enrollment in cancer clinical trials. JAMA Netw Open. 2020;3(6):e2010651. 32478845 10.1001/jamanetworkopen.2020.10651 PMC7265094

[17] Fox L Wylie H Cahill F, et al. C-CRES: COVID-19 and cancer research engagement study. Paper presented at: American Society of Clinical Oncology (ASCO) Quality Care Symposium, October 9–10, 2020.

[18] Spitzer RL Kroenke K Williams JB Löwe B. A brief measure for assessing generalized anxiety disorder: the GAD-7. Archiv Int Med. 2006;166(10):1092–1097. 10.1001/archinte.166.10.1092 16717171

[19] Holdcroft A. Gender bias in research: how does it affect evidence based medicine? J Royal Soc Med. 2007;100(1):2–3. 10.1258/jrsm.100.1.2 PMC1761670 17197669

[20] Slovic P. Perception of risk. Science. 1987;236(4799):280–285. 3563507 10.1126/science.3563507

[21] Gerhold L. COVID-19: risk perception and coping strategies. PsyArXiv; 2020.

[22] Jordan RE Adab P Cheng KK. Covid-19: risk factors for severe disease and death. BMJ. 2020;368:m1198. 32217618 10.1136/bmj.m1198

[23] Lohiniva A-L Sane J Sibenberg K Puumalainen T Salminen M. Understanding coronavirus disease (COVID-19) risk perceptions among the public to enhance risk communication efforts: a practical approach for outbreaks, Finland, February 2020. Eurosurveillance. 2020;25(13):2000317. 10.2807/1560-7917.ES.2020.25.13.2000317 PMC7140598 32265008

[24] Moghanibashi-Mansourieh A. Assessing the anxiety level of Iranian general population during COVID-19 outbreak. Asian J Psychiatry. 2020;51:102076. 10.1016/j.ajp.2020.102076 PMC7165107 32334409

[25] Liu D Ren Y Yan F, et al. Psychological impact and predisposing factors of the Coronavirus Disease 2019 (COVID-19) pandemic on general public in China (3/7/2020). Published 2020. https://ssrn.com/abstract=3551415.

[26] Wang Y Di Y Ye J Wei W. Study on the public psychological states and its related factors during the outbreak of coronavirus disease 2019 (COVID-19) in some regions of China. Psychol Health Med. 2020;26(1):13–22. 32223317 10.1080/13548506.2020.1746817

[27] Zhou S-J Zhang L-G Wang L-L, et al. Prevalence and socio-demographic correlates of psychological health problems in Chinese adolescents during the outbreak of COVID-19. Eur Child Adolesc Psychiatry. 2020;29(6):749–758. 32363492 10.1007/s00787-020-01541-4 PMC7196181

[28] McLean CP Anderson ER. Brave men and timid women? A review of the gender differences in fear and anxiety. Clin Psychol Rev. 2009;29(6):496–505. 19541399 10.1016/j.cpr.2009.05.003

[29] Menzies RG Clarke JC. The etiology of phobias: a nonassociative account. Clin Psychol Rev. 1995;15(1):23–48.

[30] Thorpe SJ Salkovskis PM. Phobic beliefs: do cognitive factors play a role in specific phobias? Behav Res Ther. 1995;33(7):805–816. 7677718 10.1016/0005-7967(95)00022-p

[31] Stone R Cafferata GL Sangl J. Caregivers of the frail elderly: a national profile. The Gerontologist. 1987;27(5):616–626. 2960595 10.1093/geront/27.5.616

[32] Kang SJ Jung SI. Age-related morbidity and mortality among patients with COVID-19. Infect Chemother. 2020;52(2):154–164. 32537961 10.3947/ic.2020.52.2.154 PMC7335648

[33] Unger JM Hershman DL Till C, et al. “When offered to participate”: a systematic review and meta-analysis of patient agreement to participate in cancer clinical trials. Paper presented at: American Society of Clinical Oncology (ASCO) Quality Care Symposium; October 9–10, 2020.

[34] Saini KS de Las Heras B de Castro J, et al. Effect of the COVID-19 pandemic on cancer treatment and research. Lancet Haematol. 2020;7(6):e432–e435. 32339482 10.1016/S2352-3026(20)30123-X PMC7195053

[35] Karzai F Madan RA Dahut W. The world of clinical trial development post-COVID-19: lessons learned from a global pandemic. Clin Cancer Res. 2020;26(16):4198–4200. 32503806 10.1158/1078-0432.CCR-20-1914

[36] West H. Telemedicine in oncology: delivering on an overdue promise in the COVID-19 era. Front Oncol. 2020;10(1870):578888. 33102236 10.3389/fonc.2020.578888 PMC7546201

[37] UK Office for National Statistics (ONS). Updating ethnic contrasts in deaths involving the coronavirus (COVID-19), England and Wales: deaths occurring 2 March to 28 July 2020. 2020.

