# Supplementary material for: Gender Differences in Concerns About Participating in Cancer Research During the COVID-19 Pandemic
Source: Cancer Control. 2021 Jan 25;28:1073274821989315. doi: 10.1177/1073274821989315 (PMC8482716; doi:10.1177/1073274821989315)
Supplement: Supplemental Material, sj-pdf-1-ccx-10.1177_1073274821989315 - Gender Differences in Concerns About Participating in Cancer Research During the COVID-19 Pandemic [file sj-pdf-1-ccx-10.1177_1073274821989315.pdf]

C-CRES: COVID-19 and Cancer Research Engagement Study

Appendix: Questionnaire

The following questions are designed to help us to understand how engagement with cancer research is being impacted by the Covid-19 outbreak.

Please try to answer all of the questions – you will not be identifiable from your responses.

The questions will take approximately 15 minutes to answer.

Q1 Which country do you live in?

[List all countries]

Q2 What was your gender assigned at birth?

Female

Male

Prefer not to say

Q3 Which category best describes your ethnicity or race?

Arab

Black (of African descent)

Black (of Caribbean descent)

Chinese

East or Southeast Asian

Latin American

Native/Indigenous

South Asian

White

Mixed ethnicity/race

Other

Prefer not to say

Q4 What is your age?

18 – 29

30 – 39

40 – 49

50 – 59

60 – 69

70 – 79

80 – 89

90 – 99

100+

Prefer not to say

Q5 Please select the category that best describes the type of cancer you were diagnosed with:

Bladder

Blood (e.g. leukaemia)

Brain, eye, or central nervous system

Breast

Colon, rectal, or anal

Head/neck, for example cancer of the mouth (not including oesophagus or brain)

Gynaecological (female genital organs, for example ovary or cervix)

Kidney

Liver

Lung

Lymphoid, stem cell, or related tissue

Neuroendocrine

Oesophagus or stomach

Pancreas

Prostate

Skin

Testicle, or penis

Thyroid (or other endocrine glands)

My doctors do not yet know exactly where the cancer is (unknown primary tumour)

I am unsure which category my diagnosis fits into

Q5a CONDITIONAL: if user indicates **Skin** to Q5

Were you diagnosed with a malignant melanoma?

Yes

No, it is another type of skin cancer

Q6 Are you currently undergoing a treatment for your cancer?

Yes

No

Q7 Thinking back to your most recent treatment for cancer, what type of treatment was this? (if you are currently undergoing treatment, please describe your current treatment)

Active surveillance/monitoring

Chemotherapy

Chemotherapy with radiotherapy

Hormonal treatment

Immunotherapy

Radiotherapy, brachytherapy, or high-frequency ultrasound (HIFU)

Surgery

Other treatment

I have not yet received any treatment or surveillance

Q8 Have you visited hospital for cancer care since the outbreak of Covid-19 in your country?

Yes

No

Q9 Have you ever been tested for Covid-19?

Yes

No

Q9a CONDITIONAL: if user indicates **Yes** to Q9

What were the results of your Covid-19 test?

Positive

Negative

Q10 Do you suspect that you have already had, and recovered from Covid-19, due to experiencing Covid-19 symptoms previously?

Yes, I think that is likely

No, I think that is unlikely

Q11 Are you currently experiencing symptoms associated with Covid-19?

Yes

No

Q12 Have you ever been hospitalised due to suspected Covid-19?

Yes

No

Q13 Have you been hospitalised for anything other than suspected Covid-19, since the outbreak of Covid-19 in your country?

Yes

No

Q14 Have you accessed any cancer organisation support information (online or otherwise) around your cancer and Covid-19?

Yes

No

Q15 Have you ever participated in a research study in cancer? [BRANCH QUESTION]

Yes, I have previously

Yes, I have previously but have withdrawn from the study due to Covid-19

Yes, I am currently

No

BRANCH 1: IF USER RESPONSE TO Q15 = Yes, I have previously

Q15a Please choose the option that best describes the type of cancer research study you were participating in:

The study involved taking a drug

The study was an observational study – I completed questionnaires and/or provided biological samples, but apart from this, I did not have to do anything else aside from my usual care

The study involved being interviewed, or taking part in a focus group discussion

The study involved a new method of performing surgery

The study was examining the effects of exercise/physical activity

The study was examining the effects of a diet change, or nutritional supplement

The study was examining the effects of a psychological support intervention

The study was a mixture of the above

I am unsure which option the study comes under

Q15b Did the study require you to travel somewhere to participate?

Yes

No

Q15b1 CONDITIONAL: if user indicates Yes to Q15b

Where did you need to travel to for the study?

Hospital

My local doctor/GP

My pharmacy

A community gym

A community centre

Other

Q15c If you had been invited to take part in this study after the outbreak of Covid-19 in your country, would you have felt comfortable participating?

I would feel more motivated to participate due to Covid-19

I would feel OK and would participate

I would have concerns due to Covid-19, but would be likely to participate

I would have concerns due to Covid-19 that I would need addressing by study researchers before I could participate

I would have concerns due to Covid-19, and would probably not participate for that reason

BRANCH 2: IF USER RESPONSE TO Q15 = Yes, I have previously but have withdrawn from the study due to Covid-19

Q15d Please choose the option that best describes the type of cancer research study you were participating in:

The study involved taking a drug

The study was an observational study – I completed questionnaires and/or provided biological samples, but apart from this, I did not have to do anything else aside from my usual care

The study involved being interviewed, or taking part in a focus group discussion

The study involved a new method of performing surgery

The study was examining the effects of exercise/physical activity

The study was examining the effects of a diet change, or nutritional supplement

The study was examining the effects of a psychological support intervention

The study was a mixture of the above

I am unsure which option the study comes under

Q15e Did the study require you to travel somewhere to participate?

Yes

No

Q15e1 CONDITIONAL: if user indicates Yes to Q15e

Where did you need to travel to for the study?

Hospital

My local doctor/GP

My pharmacy

A community gym

A community centre

Other

Q15e2 CONDITIONAL: if user indicates Yes to Q15e

How would you normally travel to participate in the study?

Personal car

Bus

Taxi

Train

Bicycle

Walk

Other mode of transport

BRANCH 3: IF USER RESPONSE TO Q15 = Yes, I am currently

Q15f Please choose the option that best describes the type of cancer research study you are involved in:

The study involves taking a drug

The study is an observational study – I complete questionnaires and/or provide biological samples, but apart from this, I do not have to do anything else aside from my usual care

The study involves being interviewed, or taking part in a focus group discussion

The study involves a new method of performing surgery

The study is examining the effects of exercise/physical activity

The study is examining the effects of a diet change, or nutritional supplement

The study is examining the effects of a psychological support intervention

The study is a mixture of the above

I am unsure which option the study comes under

Q15g Does the study normally require you to travel somewhere to participate?

No

Yes

Q15g1 CONDITIONAL: if user indicates Yes to Q15g

Where would you normally need to travel to for the study?

Hospital

My local doctor/GP

My pharmacy

A community gym

A community centre

Other

Q15g2 CONDITIONAL: if user indicates Yes to Q15g

How would you normally travel to participate in the study?

Personal car

Bus

Taxi

Train

Bicycle

Walk

Other mode of transport

Q15h Has the study you are participating in been stopped (even temporarily) due to circumstances surrounding the Covid-19 outbreak?

Yes

No

Q15h1 CONDITIONAL: if user indicates Yes to Q15h

Do you have an idea of when the study will start again?

Yes

No

The study has been cancelled entirely

Q15h2 CONDITIONAL: if user indicates Yes to Q15h

If the study were to resume, would you feel comfortable continuing to participate in the study during the current Covid-19 pandemic?

I feel more motivated to participate due to Covid-19

I would feel OK and would continue to participate

I would have concerns due to Covid-19, but would be likely to participate anyway

I would have concerns due to Covid-19 that I would need addressing by study researchers before I could continue to participate

I would have concerns due to Covid-19, and would probably not continue to participate for that reason

Q15h3 CONDITIONAL: if user indicates No to Q15h

Have the study researchers asked you to follow a different procedure due to Covid-19, to allow for the study data collection to continue?

Yes

No

I'm not sure

Q15h4 CONDITIONAL: if user indicates No to Q15h

Do you feel comfortable continuing to participate in the study during the current outbreak of Covid-19?

I feel more motivated to participate because of Covid-19

I feel OK and will continue to participate if possible

I have concerns due to Covid-19, but will continue to participate anyway if possible

I have concerns due to Covid-19 that I would like addressing by study researchers, so that I can continue to participate

I have concerns due to Covid-19, and am considering withdrawing from the study for that reason

BRANCH 4: IF USER RESPONSE TO Q15 = No

Q15i Would you feel comfortable participating in a cancer research study during the current Covid-19 pandemic?

I would feel more motivated to participate because of Covid-19

I would feel OK and would participate if the study interested me

I would have concerns due to Covid-19, but would be likely to participate anyway, if the study interested me

I would have concerns due to Covid-19 that I would need addressing / resolving by study researchers, before I could participate

I would have concerns due to Covid-19, and would not participate for that reason

Q15j Do you normally have to travel somewhere for your cancer care?

Yes

No

Q15j1 CONDITIONAL: if user indicates Yes to Q15j

How would you normally travel for your cancer care?

Personal car

Bus

Taxi

Train

Bicycle

Walk

Other mode of transport

Q16 Do you live alone?

Yes

No

Q16a CONDITIONAL: if user indicates No to Q16

Do you live with someone who is 'shielding'/isolating themselves from those outside of their household, due to increased risk to them from Covid-19?

Yes

No

Q16b CONDITIONAL: if user indicates No to Q16

Do you live with someone who you personally think is at increased risk from Covid-19? (but not necessarily 'shielding'/isolating themselves from others)

Yes

No

Q16c CONDITIONAL: if user indicates No to Q16

Do you have dependent children?

Yes

No

Q17 Do you have caring responsibilities? (not including childcare responsibilities)

Yes

No

Q18 Do you have any of the following medical conditions? (please tick all that apply)

Hypertension (high blood pressure)

Diabetes (either Type 1 or Type 2)

Any lung conditions

Any kidney conditions

Any liver conditions

Heart disease

Long term use of steroid medication

None of the above

Q19 If you are working at the moment, how difficult is it to physically distance (stay 1 or 2 metres apart) from others when at work?

Not difficult

Somewhat difficult

It is impossible

I am not working at the moment

Q20 Please indicate how concerning each of the following things are to you, with regards to taking part in cancer research during the current Covid-19 pandemic:

	Not at all concerned	Mildly concerned	Moderately concerned	Seriously concerned
My age				
My (non-cancer) medical conditions				
My occupation/job				
My ethnicity/race				
Financial/health insurance				
The type of research study that I am taking part in				
Having to travel somewhere to take part in research				
The type of travel that I have to use to take part (e.g. bus, car, train)				
Those that I live with				
Those that I have caring responsibilities toward (not including childcare)				
Cancer treatment that I am currently undergoing				
Cancer treatment that I have previously undergone				
The type of cancer that I have been diagnosed with				
Other (please specify in next question)				

Q21 If you indicated 'Other' concerns in the previous question, what are they? Please take care not to write anything in your answer which could be used to identify you.

[Free text box]

Q23 Over the last 2 weeks, how often have you been bothered by any of the following problems?

	Not at all	Several days	More than half the days	Nearly every day
Feeling nervous, anxious, or on edge				
Not being able to stop or control worrying				
Worrying too much about different things				
Trouble relaxing				
Being so restless that it is hard to sit still				
Becoming easily annoyed or irritable				
Feeling afraid as if something awful might happen				
